# Differences between two sodium hyaluronate-based submucosal injection materials currently used in Japan based on viscosity analysis

**DOI:** 10.1038/s41598-021-85118-4

**Published:** 2021-03-11

**Authors:** Ryohei Hirose, Takuma Yoshida, Yuji Naito, Naoto Watanabe, Hikaru Hashimoto, Satoshi Sugino, Risa Bandou, Tomo Daidoji, Ken Inoue, Osamu Dohi, Naohisa Yoshida, Takaaki Nakaya, Yoshito Itoh

**Affiliations:** 1 Department of Molecular Gastroenterology and Hepatology, Graduate School of Medical Sciences, Kyoto Prefectural University of Medicine, 465 Kajii-cho, Kawaramachi-Hirokoji, Kamigyo-ku, Kyoto, 602-8566 Japan; 2 Department of Infectious Diseases, Graduate School of Medical Sciences, Kyoto Prefectural University of Medicine, Kyoto, Japan

**Keywords:** Biomaterials, Gastroenterology, Gastrointestinal diseases

## Abstract

In Japan, two 0.4% sodium hyaluronate (HA)-based submucosal injection materials (SIMs) are currently used in endoscopic submucosal dissection (ESD): MucoUp (HA-Mc) and Ksmart (HA-Ks). HA-Mc and HA-Ks have the same concentration and are, thus, construed by most endoscopists to have no difference. Nevertheless, visual observation conveys the impression that HA-Ks have a higher viscosity than HA-Mc, suggesting that HA-Ks performs better than HA-Mc. This study aimed to examine the differences between HA-Mc and HA-Ks. HA-Ks exhibited higher viscosity due to greater weight-average molecular weight compared with HA-Mc. HA-Ks had significantly greater submucosal elevation height (SEH) than HA-Mc; the SEH of HA-Ks-80% (80% dilution of HA-Ks) was the same as that of HA-Mc. The ESD procedure time was significantly shorter with HA-Ks than with HA-Mc (15.2 ± 4.1 vs. 19.5 ± 5.9; *P* = 0.049). The total injection volume for HA-Ks was significantly lower than that for HA-Mc (10.8 ± 3.6 vs. 14.4 ± 4.6; *P* = 0.045). However, no significant difference in these items was observed between HA-Mc and HA-Ks-80%. HA-Mc and HA-Ks were considered to be almost the same. Nonetheless, HA-Ks exhibited higher viscosity and SIM performance than HA-Mc. HA-Ks-80% had almost the same performance as HA-Mc. Thus, understanding SIM performance and characteristics requires a focus on the viscosity of SIMs.

## Introduction

Endoscopic submucosal dissection (ESD) is performed for gastrointestinal cancer or polyp in several medical institutions, and the number of endoscopic treatments is increasing year by year^1–6^. The use of high-performance submucosal injection materials (SIMs) can strongly support the realization of rapid and safe endoscopic treatments^7–9^. High-performance SIMs currently used in endoscopic treatments are viscous solutions, and recent studies have described the viscoelastic properties of solutions suitable for high-performance SIMs^7–11^.

SIMs based on sodium hyaluronate (HA) were developed in around 2000 and are currently widely used in endoscopic treatments owing to their superior performance and low tissue toxicity^12–14^. MucoUp (Boston Scientific, Marlborough, MA, USA) and Ksmart (Olympus, Tokyo, Japan) are the two HAs currently marketed and used in Japan, and both have the same HA concentration of 0.4%. HA-Ks (Ksmart) began to be used in 2019—that is, more than 10 years after the launch of HA-Mc (MucoUp). As HA-Mc and HA-Ks have the same HA concentration, most endoscopists construe that HA-Mc practically does not differ from HA-Ks with respect to viscosity, ease of injection, and SIM performance. Therefore, both HA-Mc and HA-Ks are being used and regarded as the same SIM in actual endoscopic treatment.

Visual observation conveys the impression that there exists a clear difference in viscosity between HA-Mc and HA-Ks. Specifically, the flow speed of the solution and the disappearance speed of bubbles generated in the solution are clearly lower in HA-Ks than in HA-Mc (Video 1), indicating that HA-Ks has higher viscosity than HA-Mc. Even if the HA concentration is the same, HA solutions with different weight-average molecular weights may have different viscosities^15–17^. Hence, consistent with this visual observation, it is quite possible that HA-Mc differs from HA-Ks with respect to viscosity. Our previous studies showed that a SIM with higher viscosity has higher performance, as viscosity is strongly positively correlated to performance^10,11^. Based on these findings, HA-Ks is expected to have higher SIM performance than HA-Mc.

Through detailed evaluations, the present study aimed to examine the differences between HA-Mc and HA-Ks and to demonstrate that understanding SIM performance and characteristics requires a focus on the components, concentrations, and viscosity of SIMs. We developed original methods for the accurate measurements of viscosity, submucosal elevation height (SEH), and injection pressure (IP) of SIMs^11,18,19^. Utilizing these methods, we precisely evaluated the viscosity and SIM performance of HA-Mc and HA-Ks. Furthermore, the weight-average molecular weights of HA in HA-Mc and HA-Ks were compared.

## Results

### Viscosity analysis

**Figure 1:**
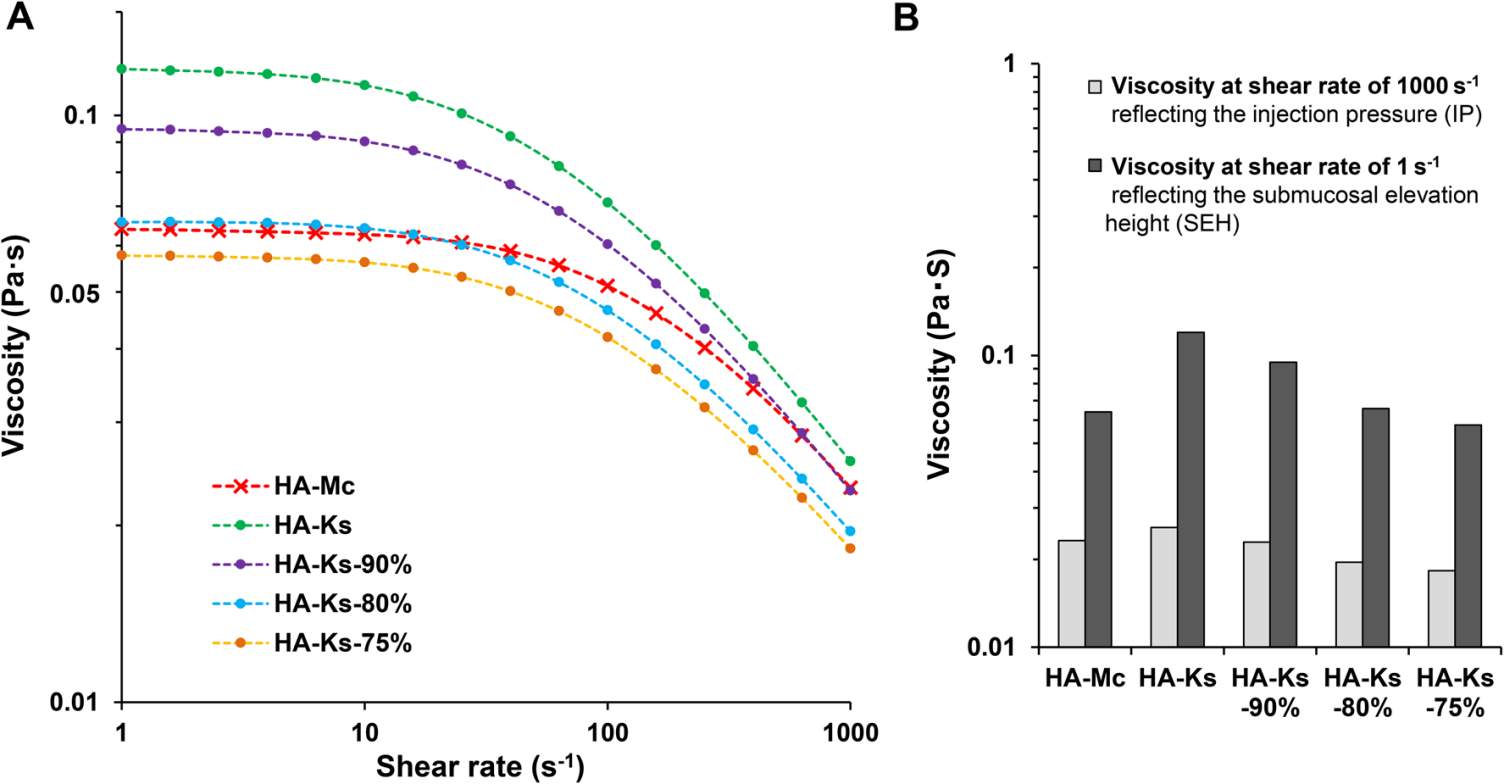
Viscosity analysis for HA-Mc and HA-Ks. (**A**) Viscosity of HA-Mc, HA-Ks, HA-Ks-90%, HA-Ks-80%, and HA-Ks-75% at each shear rate. (**B**) Comparison of viscosity between HA-Mc and HA-Ks at shear rates of 1 s^−1^ and 1000 s^−1^. Viscosity at shear rates of 1 s^−1^ and 1000 s^−1^ most significantly affected the submucosal elevation height and injection pressure, respectively. HA-Mc, sodium hyaluronate-based submucosal injection material (MucoUp); HA-Ks, sodium hyaluronate-based submucosal injection material (Ksmart).

A viscosity analysis was conducted for the fluid-flow properties of HA-Mc and HA-Ks. Both HA-Mc and HA-Ks exhibited the characteristics of a Newtonian fluid (i.e., a fluid with a viscosity that does not change even if the shear rate changes) at low shear rates (0.01–1 s^−1^) and, to a slight extent, the characteristics of a pseudoplastic fluid (i.e., a fluid with a viscosity that decreases as the shear rate increases) at high shear rates (1–100 s^−1^). HA-Ks showed a greater decrease in viscosity with increasing shear rate than HA-Mc, indicating stronger pseudoplastic properties. The viscosity of HA-Ks was higher than that of HA-Mc at all shear rates (0.01–100 s^−1^; Fig. 1A).

In our previous study, viscosity at a shear rate of 1 s^−1^ most significantly affected the SIM performance (i.e., SEH), and viscosity at a shear rate of 1000 s^−1^ most significantly affected the ease of injection (i.e., IP). Therefore, viscosity evaluation was performed with emphasis on these two values. At a shear rate of 1 s^−1^, the viscosity of HA-Ks was significantly higher than that of HA-Mc (0.120 ± 0.006 vs. 0.064 ± 0.005; *P* < 0.001), and the viscosities of HA-Ks-80% and HA-Mc were almost the same (0.066 ± 0.002 vs. 0.064 ± 0.005; *P* = 0.627). This result indicated that HA-Ks had higher SIM performance than HA-Mc and that HA-Ks-80% and HA-Mc had almost the same SIM performance. At a shear rate of 1000 s^−1^, the viscosity of HA-Ks was slightly higher than that of HA-Mc (0.026 ± 0.002 vs. 0.023 ± 0.001; *P* = 0.065), whereas the viscosity of HA-Ks-80% was slightly lower than that of HA-Mc (0.020 ± 0.001 vs. 0.023 ± 0.001; *P* < 0.001), suggesting that HA-Mc had a slightly lower IP than HA-Ks but a slightly higher IP than HA-Ks-80% (Fig. 1B).

### Comparison of weight-average molecular weight between HA-Mc and HA-Ks

**Figure 2:**
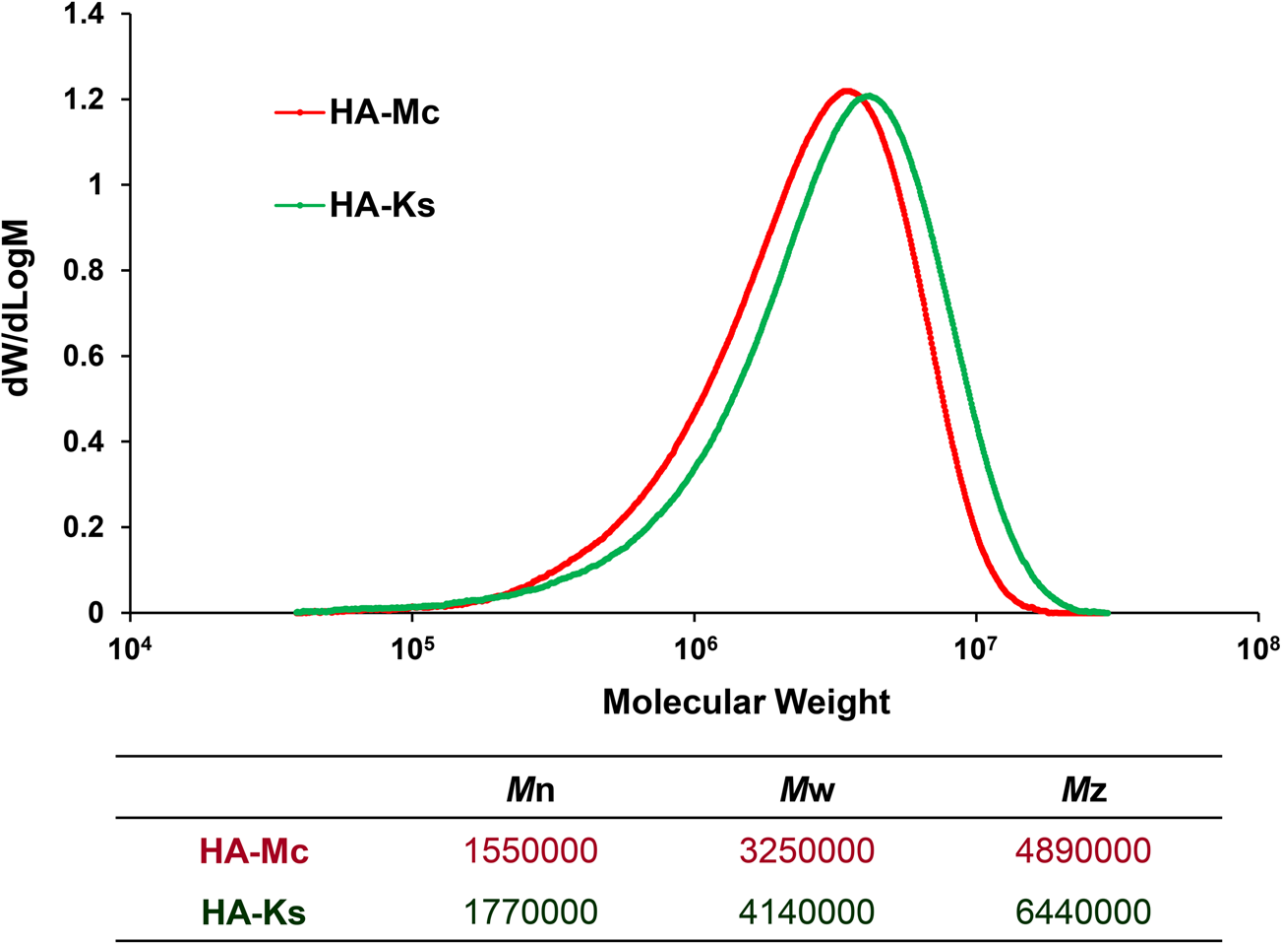
Comparison of average molecular weight between HA-Mc and HA-Ks. Molecular weight distributions of HA-Mc and HA-Ks were measured using size exclusion chromatography (SEC), and molecular weight distribution curves were created. Mw represents the weight-average molecular weight; Mn, the number average molecular weight; and Mz, the Z-average molecular weight. The weight-average molecular weight (Mw) has the strongest correlation with physical properties (especially viscosity). HA-Mc, sodium hyaluronate-based submucosal injection material (MucoUp); HA-Ks, sodium hyaluronate-based submucosal injection material (Ksmart).

Molecular weight distributions of HA-Mc and HA-Ks were measured by size exclusion chromatography (SEC), and molecular weight distribution curves were created. The comparison of molecular weight distributions between HA-Mc and HA-Ks revealed that HA-Ks had higher molecular weight than HA-Mc. Furthermore, the weight-average molecular weight, which has the strongest correlation with physical properties (especially viscosity), was approximately 1.3 times higher in HA-Ks than in HA-Mc (Fig. 2).

### SEH measurements (performance evaluation)

**Figure 3:**
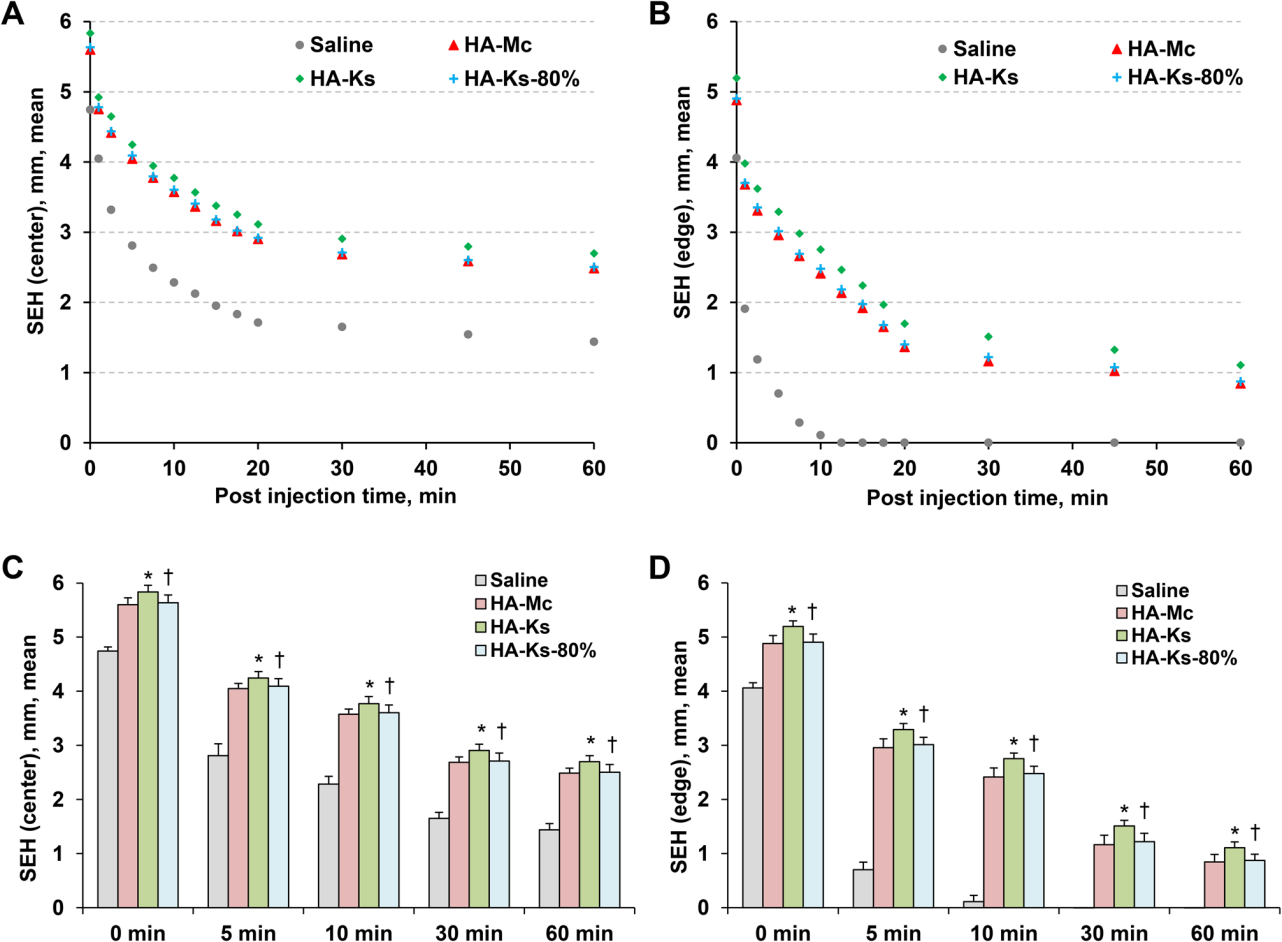
Comparison of submucosal elevation height (SEH) between saline, HA-Mc, HA-Ks, and HA-Ks-80%. (**A**) After injecting saline, HA-Mc, HA-Ks, or HA-Ks-80% into the submucosa at the center of specimens, the SEH was measured over time. (**B**) Saline, HA-Mc, HA-Ks, or HA-Ks-80% was injected at the edge of specimens instead of their center, and the SEH was measured over time. (**C, D**) Values of SEH measured at various post-injection times for saline, HA-Mc, HA-Ks, or HA-Ks-80% after injections at the center (**C**) and edge (**D**) of specimens are compared. SEH, submucosal elevation height; HA-Mc, sodium hyaluronate-based submucosal injection material (MucoUp); HA-Ks, sodium hyaluronate-based submucosal injection material (Ksmart); HA-Ks-80%, HA-Ks diluted to 80% concentration in saline. Data are expressed as mean ± standard deviations for at least five independent experiments. *P < 0.05 (HA-Mc vs. HA-Ks). ^†^*P* > 0.05 (HA-Mc vs. HA-Ks-80%).

First, each SIM was injected into the submucosa at the center of the specimens tested, and the SEH was measured over time. The SEHs of HA-Mc, HA-Ks, and HA-Ks-80% were greater than those of saline. Furthermore, the SEHs of HA-Ks were all greater than those of HA-Mc, and the SEHs of HA-Ks-80% and HA-Mc were almost the same (Fig. 3A).

Next, saline, HA-Mc, HA-Ks, and HA-Ks-80% were injected at the edge of specimens. The obtained SEH values were lower than the magnitudes measured after injection into the center of specimens; nevertheless, similar trends were observed (Fig. 3B).

Finally, the SEHs of HA-Mc and HA-Ks were compared. When HA-Mc was injected into the submucosa at the center of specimens, the SEH was 5.60 ± 0.13 mm immediately after injection and 2.69 ± 0.10 mm after 30 min. When HA-Ks was injected, the SEH was 5.83 ± 0.13 mm immediately after injection and 2.91 ± 0.11 mm after 30 min. At all post-injection times (0, 5, 10, and 30 min), the SEHs of HA-Ks were significantly greater than those of HA-Mc (*P* < 0.05; Fig. 3C and Supplementary Table 1). Moreover, at all post-injection times, no significant difference in the SEHs was noted between HA-Mc and HA-Ks-80%.

**Table 1:** Comparison of ESD outcomes in an ex vivo porcine stomach model.

	HA-Mc (n = 12)	HA-Ks (n = 12)	HA-Ks-80% (n = 12)	*P (HA-Mc vs. HA-Ks)*	*P (HA-Mc vs. HA-Ks-80%)*
ESD procedure time (min)	19.5 ± 5.9	15.2 ± 4.1	18.1 ± 9.0	0.049	0.657
Total volume of injected SIM (ml)	14.4 ± 4.6	10.8 ± 3.6	13.7 ± 5.9	0.045	0.745
Total number of SIM injection	2.3 ± 0.5	1.6 ± 0.5	1.6 ± 0.7	0.003	0.009
Ease of dissection (assessed with a 100 mm VAS)	45.0 ± 8.4	41.0 ± 8.95	41.7 ± 17.4	0.272	0.556

Statistics presented as mean ± SD with t-tests.

ESD, endoscopic submucosal dissection; VAS, visual analog scale; HA-Mc, sodium hyaluronate-based submucosal injection material (MucoUp®); HA-Ks, sodium hyaluronate-based submucosal injection material (Ksmart®); HA-Ks-80%, HA-Ks diluted to 80% concentration in saline.

Similar results were obtained when each SIM was injected into the submucosa at the edge of specimens. The SEHs of HA-Ks were significantly greater than those of HA-Mc (*P* < 0.05; Fig. 3D and Supplementary Table 2).

### IP measurements

**Figure 4:**
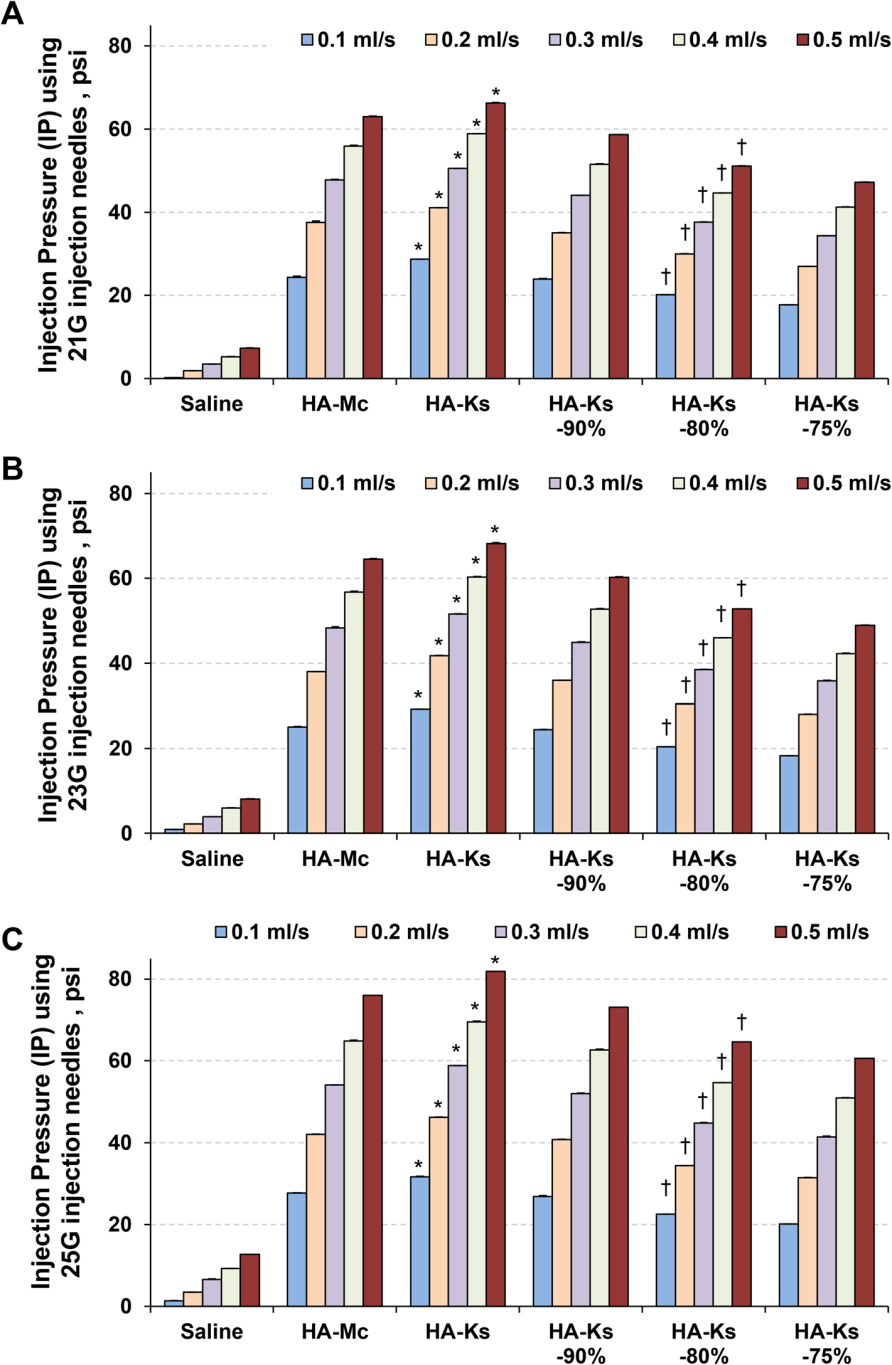
Injection pressure (IP) of saline, HA-Mc, HA-Ks, HA-Ks-90%, HA-Ks-80%, and HA-Ks-75%. (**A**) With a 21-gauge endoscopic injection needle, the IP of saline, HA-Mc, HA-Ks, HA-Ks-90%, HA-Ks-80%, and HA-Ks-75% was measured at an injection speed rate of 0.1, 0.2, 0.3, 0.4, or 0.5 mL/s. (**B**) Using a 23-gauge endoscopic injection needle, a similar IP measurement was performed. (**C**) Using a 25-gauge endoscopic injection needle, a similar IP measurement was performed. IP, injection pressure; HA-Mc, sodium hyaluronate-based submucosal injection material (MucoUp); HA-Ks, sodium hyaluronate-based submucosal injection material (Ksmart). Data are expressed as mean ± standard deviations for at least three independent experiments. *P < 0.001 (HA-Mc vs. HA-Ks). ^†^*P* < 0.001 (HA-Mc vs. HA-Ks-80%).

The IP of saline, HA-Mc, HA-Ks, HA-Ks-90%, HA-Ks-80%, and HA-Ks-75% was measured using a 21-, 23-, or 25-gauge endoscopic injection needle at an injection speed rate of 0.1, 0.2, 0.3, 0.4, or 0.5 mL s^−1^ (Supplementary Table 3). The IP increased with an increase in injection speed and a decrease in endoscopic injection needle diameter. In all conditions of injection speed and endoscopic injection needle diameter, the IP of HA-Ks injection was slightly higher than that of HA-Mc injection, and the IP of HA-Ks-80% injection was lower than that of HA-Mc injection (*P* < 0.001 for all; Fig. 4).

### ESD outcomes in ex vivo porcine stomach model

ESD was performed with HA-Mc, HA-Ks, or HA-Ks-80% in an ex vivo porcine stomach model, and the outcomes were compared (Table 1). The ESD procedure time was significantly shorter with HA-Ks than with HA-Mc (15.2 ± 4.1 vs. 19.5 ± 5.9; *P* = 0.049). The total injection volume was significantly lower for HA-Ks than for HA-Mc (10.8 ± 3.6 vs. 14.4 ± 4.6; *P* = 0.045). The total number of submucosal HA-Ks injections per treatment was considerably smaller than the total number of submucosal HA-Mc injections (1.6 ± 0.5 vs. 2.3 ± 0.5; *P* = 0.003). No significant difference in visual analog scale (VAS) measurements in ESD (41.0 ± 8.95 vs. 45.0 ± 8.4; *P* = 0.272) was observed between ESD using HA-Ks and ESD using HA-Mc. Furthermore, there were no significant differences between HA-Mc and HA-Ks-80% with respect to ESD procedure time, total volume of injected SIM, and VAS measurements in ESD.

## Discussion

As HA-Mc and HA-Ks are HA solutions of the same concentration (0.4%), both SIMs have been considered to have almost identical characteristics: viscosity, ease of local injection (i.e., IP), and SIM performance (i.e., SEH). However, HA solutions with different weight-average molecular weights may have different viscosities even if the HA concentration is the same, and the HA solution with higher weight-average molecular weight generally tends to have higher viscosity^15–17^. Therefore, focusing on the constituent components and concentrations of SIMs and directly measuring and comparing the viscosities of SIMs are extremely important to understand the differences in SIM characteristics.

The viscosity analysis showed that the viscosity of HA-Ks was higher than that of HA-Mc at all shear rates. Furthermore, the evaluation of molecular weight distribution by SEC revealed that HA-Ks contained HA with higher weight-average molecular weight than HA-Mc. These results indicated that HA-Ks had higher viscosity due to greater weight-average molecular weight than HA-Mc. The difference in weight-average molecular weight between HA-Mc and HA-Ks is expected to be due to differences in the manufacturing process of sodium hyaluronate between the two agents. In addition, previous studies reported a strong positive correlation between SIM viscosity and SEH/IP^10,11^; hence, it was expected that HA-Ks had higher SEH and IP than HA-Mc. In actual measurements, the SEH and IP of HA-Ks were significantly higher than those of HA-Mc, suggesting that HA-Ks exhibited higher performance than HA-Mc.

In our previous study, the strongest correlations were observed between viscosity at a shear rate of 10^3^ s^−1^ and IP and between viscosity at a shear rate of 10^0^ s^−1^ and SEH^11^. The results of this study indicate that pseudoplastic fluid-based SIMs (i.e., SIMs with a viscosity that drastically decreases with increasing shear rate) are estimated to have higher performance than Newtonian fluid-based SIMs (i.e., SIMs with a constant viscosity despite changes in shear rate) when both types of SIMs have the same ease of injection^10,11,20,21^. The viscosity analysis revealed that the viscosities of HA-Ks and HA-Ks-80% tended to decrease at high shear rates, as compared to the viscosity of HA-Mc, and that HA-Ks and HA-Ks-80% exhibited stronger pseudoplastic fluid characteristics than HA-Mc. Therefore, the result of this analysis suggests that HA-Ks and HA-Ks-80% have more suitable viscosity characteristics as high-performance SIMs than HA-Mc. In fact, the viscosity at a shear rate of 10^0^ s^−1^ and SEH of HA-Ks-80% were almost the same as those of HA-Mc, and the viscosity at a shear rate of 10^3^ s^−1^ and IP of HA-Ks-80% were lower than those of HA-Mc. In other words, HA-Ks-80% has the same SIM performance as HA-Mc and is easier to inject than HA-Mc.

In order to evaluate the SIM performance in actual endoscopic treatment, we conducted a trial using an ex vivo model of ESD to compare the results of ESD treatment with HA-Mc, HA-Ks, and HA-Ks-80%. The ESD procedure time was significantly shorter with HA-Ks than with HA-Mc, and the total injection volume was significantly lower for HA-Ks than for HA-Mc. We speculate that significantly higher SEHs of HA-Ks can reduce ESD procedure time. In the evaluation of procedure difficulty by endoscopists using VAS, no significant difference between these two SIMs was identified. These results suggest that the difference in procedure difficulty was not so great that the endoscopists could not clearly perceive it. In fact, the difference remained unnoticed by endoscopists who had used these SIMs from the time that the two SIMs were launched until the present. However, these results also indicate that HA-Ks is certainly a better SIM with higher performance than HA-Mc, and the change from HA-Mc to HA-Ks may improve the ESD outcomes. Furthermore, no significant differences in the ESD procedure time and total volume of injected SIM were noted between HA-Mc and HA-Ks-80%. These results suggest that HA-Ks-80% has almost the same SIM performance as HA-Mc. Because HA-Ks-80% has a lower infusion pressure than HA-Mc and is easier to inject than HA-Mc, considering all factors, HA-Ks-80% may be concluded to be slightly superior to HA-Mc.

From the above, our study findings suggest that changing the SIM used for ESD from HA-Mc to HA-Ks may lead to improvements in ESD outcomes. Nonetheless, the present study has some limitations that need to be underlined. This study is a basic research using an ex vivo model of ESD, and SIM performance is not the only determinant of ESD outcomes. Considering these limitations, future clinical studies are required to confirm whether the results of this study can be applied to actual ESD.

Furthermore, this study suggested that HA-Ks-80% had almost the same performance as HA-Mc and could be used for ESD instead of HA-Mc. Both HA-Mc and HA-Ks have almost the same drug price (approximately 72 USD) and the same content (20 mL)^22,23^. As HA-Ks-80% is 80% dilution of HA-Ks, the content of HA-Ks-80% is increased from 20 to 25 mL at the same drug price. This is synonymous with a 20% drop in the drug price, which suggests that the use of HA-Ks-80% may reduce medical costs without deteriorating the ESD outcomes.

Thus, the results of this study demonstrate that understanding SIM performance and characteristics requires a focus on the components, concentrations, and viscosity of SIMs. These results are applicable not only to HA but also to all viscous SIMs used worldwide and will considerably contribute to advancements in endoscopic treatments for gastrointestinal tumors via the identification of the optimal SIMs for these treatments.

In conclusion, although HA-Mc and HA-Ks were considered to be almost the same, the detailed viscosity analysis and SIM performance evaluation revealed that HA-Ks exhibited higher viscosity and SIM performance than HA-Mc. Furthermore, our study showed that HA-Ks-80% had almost the same performance as HA-Mc and could be used as an alternative to HA-Mc for ESD. Our study findings suggest that changing the SIM used for ESD from HA-Mc to HA-Ks may lead to improvements in ESD outcomes and that the use of HA-Ks-80% may reduce medical costs without deteriorating the ESD outcomes.

## Methods

### SIM preparation

Otsuka Normal Saline (Otsuka Pharmaceutical Factory, Tokushima, Japan) was used as 0.9% (w/v) sodium chloride solution (saline), whereas MucoUp and Ksmart were used as HA-Mc and HA-Ks, respectively. Additionally, Ksmart was diluted to a concentration of 90%, 80%, or 75% in saline and used as HA-Ks-90%, HA-Ks-80%, or HA-Ks-75%, respectively.

### Viscosity analysis of SIMs

Viscosity analysis was performed using a rheometer^11,24,25^. The viscosity of SIMs was measured at 25 °C using a DHR-1 controlled-stress rheometer (TA Instruments, New Castle, DE, USA) with a 60-mm cone-plate geometry (1°). A solvent trap containing distilled water prevented sample dehydration during measurement. Each SIM (1.5 mL) was loaded onto the rheometer and left for 5 min to enable relaxation to the original gel structure. Steady-flow viscosity was measured in the flow-sweep mode (steady-flow measurement) and calculated as shear stress divided by shear rate. Steady-flow viscosity (in Pascal-seconds) and shear stress (in Pascals) were determined using the TRIOS software version 4.4.0.41651 (TA Instruments) for a range of shear rates (0.01, 0.016, 0.025, 0.04, 0.063, 0.1, 0.16, 0.25, 0.4, 0.63, 1.0, 1.6, 2.5, 4.0, 6.3, 10, 16, 25, 40, 63, 100, 160, 250, 400, 630, and 1000 s^−1^).

### Measurement of weight-average molecular weight

Molecular weight distributions of HA-Mc and HA-Ks were measured using SEC, and the average molecular weights were compared. The method for measuring molecular weight distributions by SEC is described below^26–29^. Briefly, 4.9 mL of 0.1 M aqueous sodium chloride solution was added as a solvent to 0.1 mL of the measurement sample. The mixture was gently stirred at 25 °C, and filtration was subsequently performed using a 0.5-μm filter. SEC was carried out using a differential refractive index detector (RID-20A; Shimadzu, Kyoto, Japan) and TSKgel GMPWXL column (7.8 mm × 30 cm; Tosoh, Tokyo, Japan). Monodisperse pullulan (Sigma-Aldrich, St. Louis, MO, USA) was used as a standard sample. Data processing was performed using a data processing system for gel permeation chromatography (Toray Research Center, Tokyo, Japan), and molecular weight distribution curves for both samples were prepared from the measured values. The molecular weight was a relative value based on pullulan.

From these measured data, the average molecular weight was determined using the following equations:

1 $${\text{Mn}} =\Sigma \left( {{\text{Ni}} \cdot {\text{Mi}}} \right)/\Sigma {\text{Ni}}$$

2 $${\text{Mw}} =\Sigma \left( {{\text{Ni}} \cdot {\text{Mi}}^{2} } \right)/\Sigma \left( {{\text{Ni}} \cdot {\text{Mi}}} \right)$$

3 $${\text{Mz}} =\Sigma \left( {{\text{Ni}} \cdot {\text{Mi}}^{3} } \right)/\Sigma \left( {{\text{Ni}} \cdot {\text{Mi}}^{2} } \right)$$

In the abovementioned equations, Mi represents the molecular weight at each elution position; Ni, the number of molecules; Mw, the weight-average molecular weight; Mn, the number average molecular weight; and Mz, the Z‐average molecular weight. The weight-average molecular weight (Mw) has the strongest correlation with physical properties. The number average molecular weight (Mn) is the average molecular weight associated with the low-molecular-weight component, whereas the Z‐average molecular weight (Mz) is the average molecular weight associated with the high-molecular-weight component.

### Evaluation of SIM performance (SEH measurement)

We previously developed a new ex vivo model that could accurately measure the SEH after SIM injection^18–20^. In the present study, the same ex vivo model was used to evaluate SIM performance. Specifically, gastric specimens were cut into 5 × 5 cm squares and immediately stored at − 30 °C. To ensure uniform conditions, all frozen gastric specimens were thawed just before the analysis procedure. Thawed specimens were stretched flat on a rubber board, and a constant tension (1.5 N) was applied by stretching both ends of the specimens with a clip.

To evaluate the performance of various SIMs, the magnitudes of SEH were measured at specific time intervals. With a 2.5-mL syringe and 23-gauge needle, 2.5 mL of each solution was horizontally injected into the submucosa at the center and edge of specimens to produce submucosal elevation. SEH was precisely measured using a digital height gauge (HDS-20C; Mitutoyo, Kanagawa, Japan) at 0, 2.5, 5, 7.5, 10, 12.5, 15, 17.5, 20, 30, 45, and 60 min after injection.

Injection into the submucosal layer at the edge of specimens reproduced the conditions of submucosal injection during endoscopic treatments after mucosal incision (such as the process of submucosal resection during ESD). Five independent measurements were performed, and the obtained results were expressed as means and standard deviations.

### Evaluation of the ease of submucosal injection (IP measurement)

We previously established a method for accurate IP measurement^11^. To evaluate the IP of each SIM, a 21-, 23-, or 25-gauge endoscopic injection needle (Needle Master; Olympus), digital pressure gauge (BN-PGD60PL-F1; Nihon Seiki, Osaka, Japan), and syringe with a solution were connected to a three-way stopcock (TS-TL1K; Terumo, Tokyo, Japan). The digital pressure gauge was connected to the three-way stopcock via an infusion extension tube. When the pressure value was stable for 3 s, the magnitudes of IP were measured using a syringe pump (Legato 100; KD Scientific, Holliston, MA, USA) that could set the injection speed and syringe type. A 20-mL or 10-mL syringe (Terumo) was used, and the injection speed was set at a rate of 0.1, 0.2, 0.3, 0.4, or 0.5 mL/s. Three independent IP measurements were performed for each SIM, and the obtained results were expressed as means and standard deviations.

### Blinded assessment of ESD using off‐label SIMs

To examine the performance of HA-Mc, HA-Ks, and HA-Ks-80%, blinded head‐to‐head comparisons of two off‐label SIMs were performed using an ex vivo porcine stomach model of ESD^20,23,30–33^. For this ex vivo model of ESD, stomachs obtained from 6-month-old domestic hybrid female pigs were immediately frozen after harvesting and thawed at 25 °C for 6 h before each experiment. The pyloric side of the stomach was fixed to an overtube (TOP Overtube; TOP, Tokyo, Japan). As the upper third of the porcine stomach is similar to the human stomach, a pseudolesion was created on the upper stomach.

ESD was conducted on a 2.5‐cm‐diameter pseudolesion in the ex vivo porcine stomach model by an endoscopist (either N.W., H.H., S.S., or T.Y.) with experience in performing around 50–100 gastrointestinal ESD procedures. All pseudolesions were created in the upper body of the porcine stomach, where good operability of the scope was ensured. The SIM used for each procedure was randomized, and the endoscopist was blinded to the SIM used; three ESD procedures per SIM were performed. The primary outcome was the total ESD procedure time. The secondary outcomes were the total volume of injected SIM, total number of SIM injections, and ease of dissection, as assessed using a 100‐mm VAS, with the highest and lowest scores indicating “extremely difficult” and “extremely easy,” respectively.

ESD was performed as follows. For this study, a gastrointestinal endoscope (GIF-Q260; Olympus) with a transparent hood was used, and the Endosaber (Sumitomo Bakelite, Tokyo, Japan) was utilized to create pseudolesions on the mucosa of the stomach model. To create a safe submucosal elevation, an appropriate amount of SIM was injected into the submucosal layer using a 25-gauge endoscopic injection needle (01,885: TOP). Subsequently, the mucosa around the pseudolesions was circumferentially incised using the Endosaber and VIO 3 electrosurgical generator (Endocut I mode, effect 2, 90 W; Erbe Elektromedizin GmbH, Tübingen, Germany). Finally, the connective tissue of the submucosal layer was dissected using a Flush knife with a swift coagulation current (effect 4, 120 W) (VIO 3; Erbe Elektromedizin GmbH). The procedure time, total volume of injected SIM, total number of SIM injection, and ease of dissection were evaluated for each ESD.

Finally, histologic sections were made from each resected block and were stained with hematoxylin and eosin. The specimen was then microscopically evaluated, and confirmed that both the ESD specimens resected using HA-Mc and HA-Ks had the submucosal layer properly dissected.

### Ethical considerations

The above-described protocols using porcine gastrointestinal tracts were conducted in accordance with the animal care guidelines of Kyoto Prefectural University of Medicine. All methods in this study were performed in compliance with the ARRIVE guidelines and the other relevant guidelines and regulations.

### Statistical analysis

For all repeated experiments, the data obtained were presented as means and standard deviations and analyzed using the GraphPad Prism 7 software (GraphPad, La Jolla, CA, USA). Continuous variables were analyzed using Student’s *t*-test. All reported *P* values were two-sided, and magnitudes with *P* < 0.05 were considered statistically significant.

## Supplementary information

Supplementary information.

Supplementary video.

## Data Availability

All data included in this study are available from the corresponding author on request.

## Abbreviations

SIM: Submucosal injection material
SHE: Submucosal elevation height
IP: Injection pressure
ESD: Endoscopic submucosal dissection
HA: Sodium hyaluronate
HA-Mc: Sodium hyaluronate-based submucosal injection material (MucoUp)
HA-Ks: Sodium hyaluronate-based submucosal injection material (Ksmart)
VAS: Visual analog scale
SEC: Size exclusion chromatography
