# Supplementary material for: Differences between two sodium hyaluronate-based submucosal injection materials currently used in Japan based on viscosity analysis
Source: Sci Rep. 2021 Mar 11;11:5693. doi: 10.1038/s41598-021-85118-4 (PMC7952736; doi:10.1038/s41598-021-85118-4)
Supplement: Supplementary file 1 — Supplementary information. [file 41598_2021_85118_MOESM1_ESM.pdf]

Supplementary material

Differences between two sodium hyaluronate-based submucosal injection materials currently used in Japan based on viscosity analysis

Ryohei Hirose,[1,2*] Takuma Yoshida,[1] Yuji Naito,[1] Naoto Watanabe,[1] Hikaru Hashimoto,[1] Satoshi Sugino,[1] Risa Bandou,[2] Tomo Daidoji,[2] Ken Inoue,[1] Osamu Dohi,[1] Naohisa Yoshida,[1] Takaaki Nakaya,[2] Yoshito Itoh[1]

[1]Department of Molecular Gastroenterology and Hepatology, Graduate School of Medical Science, Kyoto Prefectural University of Medicine, Kyoto, Japan
[2]Department of Infectious Diseases, Graduate School of Medical Science, Kyoto Prefectural University of Medicine, Kyoto, Japan

*Corresponding author: Ryohei Hirose
Department of Molecular Gastroenterology and Hepatology, Graduate School of Medical Science, Kyoto Prefectural University of Medicine, 465 Kajii-cho, Kawaramachi-Hirokoji, Kamigyo-ku, Kyoto 602-8566, Japan. Tel.: +81-75-251-5519, fax: +81-75-251-0710, e-mail: ryo-hiro@koto.kpu-m.ac.jp

Table of Contents

1. Supplementary Table 1.
2. Supplementary Table 2.
3. Supplementary Table 3.

Supplementary Table 1. Comparison of mean SEH (center) between saline, HA-Mc, HA-Ks, and HA-Ks-80%.

	Post injection time, min												
	**0**	**1**	**2.5**	**5**	**7.5**	**10**	**12.5**	**15**	**17.5**	**20**	**30**	**45**	**60**
**Saline**	4.74	4.05	3.32	2.81	2.49	2.28	2.12	1.95	1.83	1.71	1.65	1.54	1.44
**HA-Mc**	5.60	4.75	4.42	4.05	3.78	3.57	3.36	3.16	3.01	2.90	2.69	2.59	2.49
**HA-Ks**	5.83	4.92	4.65	4.25	3.95	3.77	3.57	3.38	3.25	3.11	2.91	2.80	2.70
**HA-Ks-80%**	5.63	4.78	4.44	4.09	3.80	3.60	3.41	3.18	3.02	2.92	2.71	2.60	2.50
P (HA-Mc vs. HA-Ks)	0.019	0.035	0.017	0.021	0.031	0.025	0.010	0.013	0.009	0.014	0.011	0.013	0.012
P (HA-Mc vs. HA-Ks-80%)	0.706	0.751	0.815	0.585	0.819	0.709	0.525	0.796	0.900	0.855	0.750	0.847	0.818

The values of SEH (mm) were expressed as mean and evaluated with t-test.
SEH, submucosal elevation height; HA-Mc, sodium hyaluronate-based submucosal injection material (MucoUp®); HA-Ks, sodium hyaluronate-based submucosal injection material (Ksmart®); HA-Ks-80%, HA-Ks diluted to 80% concentration in saline.

Supplementary Table 2. Comparison of mean SEH (edge) between saline, HA-Mc, HA-Ks, and HA-Ks-80%.

	**Post injection time, min**												
	**0**	**1**	**2.5**	**5**	**7.5**	**10**	**12.5**	**15**	**17.5**	**20**	**30**	**45**	**60**
**Saline**	4.06	1.91	1.19	0.70	0.29	0.11	0	0	0	0	0	0	0
**HA-Mc**	4.88	3.68	3.31	2.96	2.66	2.41	2.14	1.92	1.65	1.36	1.16	1.02	0.85
**HA-Ks**	5.20	3.98	3.62	3.29	2.98	2.75	2.46	2.24	1.97	1.70	1.51	1.32	1.11
**HA-Ks-80%**	4.90	3.70	3.35	3.01	2.69	2.48	2.18	1.98	1.68	1.40	1.22	1.08	0.87
P (HA-Mc vs. HA-Ks)	0.005	0.007	0.004	0.005	0.007	0.005	0.004	0.006	0.009	0.006	0.005	0.009	0.011
P (HA-Mc vs. HA-Ks-80%)	0.824	0.817	0.604	0.565	0.755	0.512	0.599	0.573	0.760	0.700	0.594	0.566	0.736

The values of SEH (mm) were expressed as mean and evaluated with t-test.
SEH, submucosal elevation height; HA-Mc, sodium hyaluronate-based submucosal injection material (MucoUp®); HA-Ks, sodium hyaluronate-based submucosal injection material (Ksmart®); HA-Ks-80%, HA-Ks diluted to 80% concentration in saline.

Supplementary Table 3. The injection pressure (IP) of all SIMs.

		IP, psi, mean ± SD				
		**IS = 0.1 ml/s**	**IS = 0.2 ml/s**	**IS = 0.3 ml/s**	**IS = 0.4 ml/s**	**IS = 0.5 ml/s**
**Saline**	21G	0.15 ± 0.00	1.89 ± 0.00	3.48 ± 0.00	5.22 ± 0.11	7.30 ± 0.07
	23G	0.87 ± 0.00	2.18 ± 0.00	3.92 ± 0.00	5.90 ± 0.00	8.03 ± 0.07
	25G	1.35 ± 0.07	3.48 ± 0.00	6.58 ± 0.14	9.28 ± 0.00	12.67 ± 0.07
**HA-Ms**	21G	24.37 ± 0.24	37.56 ± 0.34	47.81 ± 0.07	55.94 ± 0.18	63.04 ± 0.07
	23G	24.99 ± 0.14	38.00 ± 0.00	48.30 ± 0.24	56.76 ± 0.18	64.49 ± 0.14
	25G	27.65 ± 0.07	42.01 ± 0.07	54.10 ± 0.00	64.83 ± 0.21	76.00 ± 0.00
**HA-Ks**	21G	28.72 ± 0.00	41.05 ± 0.00	50.52 ± 0.07	58.84 ± 0.07	66.28 ± 0.12
	23G	29.20 ± 0.07	41.72 ± 0.07	51.54 ± 0.07	60.29 ± 0.07	68.17 ± 0.21
	25G	31.62 ± 0.12	46.22 ± 0.07	58.89 ± 0.00	69.47 ± 0.24	81.85 ± 0.18
**HA-Ks-90%**	21G	23.88 ± 0.14	35.05 ± 0.07	44.09 ± 0.00	51.54 ± 0.14	58.64 ± 0.07
	23G	24.41 ± 0.07	35.97 ± 0.00	44.87 ± 0.14	52.70 ± 0.14	60.24 ± 0.07
	25G	26.83 ± 0.24	40.71 ± 0.14	51.97 ± 0.14	62.66 ± 0.21	73.15 ± 0.18
**HA-Ks-80%**	21G	20.11 ± 0.07	29.93 ± 0.07	37.61 ± 0.07	44.67 ± 0.00	51.10 ± 0.07
	23G	20.31 ± 0.00	30.46 ± 0.00	38.48 ± 0.07	45.98 ± 0.00	52.79 ± 0.00
	25G	22.48 ± 0.00	34.37 ± 0.00	44.77 ± 0.14	54.68 ± 0.00	64.59 ± 0.14
**HA-Ks-75%**	21G	17.69 ± 0.00	26.98 ± 0.00	34.33 ± 0.07	41.24 ± 0.07	47.23 ± 0.07
	23G	18.27 ± 0.00	27.99 ± 0.00	35.87 ± 0.07	42.25 ± 0.14	48.88 ± 0.12
	25G	20.16 ± 0.00	31.42 ± 0.07	41.43 ± 0.18	50.91 ± 0.12	60.58 ± 0.14

IS, injection speed; HA-Mc, sodium hyaluronate-based submucosal injection material (MucoUp®); HA-Ks, sodium hyaluronate-based submucosal injection material (Ksmart®) ; HA-Ks-90%, HA-Ks diluted to 90% concentration in saline; HA-Ks-80%, HA-Ks diluted to 80% concentration in saline; HA-Ks-75%, HA-Ks diluted to 75% concentration in saline.
